# The essential neurological examination of the unconscious patient in the emergency room

**DOI:** 10.1002/brb3.1097

**Published:** 2018-08-28

**Authors:** Jochen Brich, Marius Steiert, Michel Rijntjes

**Affiliations:** ^1^ Department of Neurology and Neuroscience Medical Center University of Freiburg Freiburg Germany; ^2^ Department of Anesthesiology and Intensive Care Medicine Schwarzwald‐Baar‐Klinikum Villingen‐Schwenningen Villingen‐Schwenningen Germany

**Keywords:** coma, emergency room, neurological examination, unconsciousness

## Abstract

**Objective:**

To determine whether neurologists with long‐term experience in the emergency room are in general agreement about the essential components of the neurological examination (NE) used on unconscious patients in whom an obvious cause for coma is lacking.

**Methods:**

We surveyed 31 board‐certified practicing neurologists who regularly examine unconscious patients in the emergency room and asked them to list the specific components of the NE that they would normally choose to apply in at least 80% of cases.

**Results:**

Twenty‐seven neurologists rated 24 of 38 items as essential steps of the neurological examination of the unconscious patient, with a high level of agreement amongst survey participants.

**Conclusions:**

There was a high degree of consensus amongst the neurologists surveyed about which steps are essential for the NE of the unconscious patient. These findings provide an important source of validation for teaching this particular NE to medical students, as well as nonneurologists working in an emergency setting.

## 1 INTRODUCTION

Acute coma is characterized by the sudden development of prolonged unconsciousness and can have a variety of causes. Because this condition represents a medical emergency, quick assessment of the unconscious patient's airway, breathing, and circulation should also be accompanied by a swift neurological examination (NE) (Stevens & Bhardwaj, 2006; Stevens, Cadena, & Pineda, 2015). While the medical history and focused presenting of the patient can each provide clues about the etiology of the coma, the results of the NE can greatly facilitate this process by providing important information about the site of the underlying lesion or pathophysiological process (i.e., increased intracranial pressure, infection, etc.). This especially pertains to emergency patients who present to the hospital with a disorder of consciousness that lacks an obvious cause, such as cardiac arrest or traumatic brain injury (TBI) (Kanich et al., 2002). Such a clinical assessment is also important because the ensuing diagnostic and clinical management procedures diverge at this point, and time‐critical decisions have to be made.

Scientific publications (e.g., Stevens & Bhardwaj, 2006; Stevens et al., 2015) as well as neurology (Bender, Remi, Feddersen, & Fesl, 2012; Biller, Gruener, & Brazis, 2011; Delank & Gehlen, 2015; Fuller, 2013; Hacke, 2016; Mattle & Mumenthaler, 2015; Posner, Saper, Schiff, & Plum, 2007; Urban, 2012) or emergency medicine (Marx, Hockberger, & Walls, 2013; Tintinalli, Stapczynski, Ma, Meckler, & Cline, 2010) textbooks often describe the NE of the unconscious patient as a complex procedure that includes a multitude of elaborate features. It should be noted, however, that these appraisals are based on the opinions of just a few experts, mostly tracing back to the approaches described either by Plum and Posner, which was first published in 1966 (Plum & Posner, 1966), or C. M. Fisher's work (Fisher, 1969) published in 1969.

The most commonly applied approach to the NE aims to differentiate between focal asymmetric clinical deficits, primarily located in the motor system, and nonfocal symmetric findings; this can then help determine the underlying cause of the symptoms as being a localized structural brain lesion/functional disturbance (e.g., ischemic or epileptic) vs. nonstructural events (e.g., toxic‐metabolic), respectively (Stevens & Bhardwaj, 2006; Stevens et al., 2015). However, severe neurological diseases such as meningitis, subarachnoid hemorrhage, or basilar artery occlusion (including top of the basilar syndrome) often present with coma, either without focal asymmetric deficits, or with bilateral symmetric deficits (Caplan, 1980; Mattle, Arnold, Lindsberg, Schonewille, & Schroth, 2011; Schwarz, Egelhof, Schwab, & Hacke, 1997). Furthermore, they are often difficult to detect in native cerebral CT scans. As late recognition of these conditions is associated with a high mortality rate, the identification of clinical signs that can facilitate early clinical diagnosis and the implementation of additional diagnostic steps are essential for optimizing treatment and should ideally be completed within a few minutes.

In the emergency room (ER) of most secondary and tertiary German hospitals, either the consulting neurologist or neurologists as permanent members of the ER team routinely take over the NE of unconscious patients. Strikingly, despite the time pressure associated with such an emergency situation, there are no validated step‐by‐step protocols available for a purposeful and short but sufficient NE of a comatose patient in the ER. Such protocols are potentially even more important for physicians without a background in neurology, in cases where no neurologist is available. Indeed, without daily practice in this particular examination, some nonneurologists may feel uncertain about which examination steps to choose. As a result, they often rely on common coma scales such as the Glasgow Coma Scale (GCS) (Teasdale & Jennett, 1974) or more recently devised elaborate scoring methods such as the “Full Outline of UnResponsiveness Score” (FOUR Score) (Wijdicks, Bamlet, Maramattom, Manno, & McClelland, 2005). For teaching proposes, the “Guidelines for the Neurologic Examination in Patients with Altered Level of Consciousness” by the Neurology Clerkship Core Curriculum of the American Academy of Neurology was published in 2002 (Gelb, Gunderson, Henry, Kirshner, & Józefowicz, 2002) which resulted from a consensus between the Consortium of Neurology Clerkship Directors (CNCD) and the Undergraduate Education Subcommittee (UES) of the American Academy of Neurology (AAN). However, external validation of these guidelines is lacking.

No studies to date have attempted to identify and validate the particular components of the NE that are actually applied by neurologists experienced in examining unconscious patients who present to the ER without an obvious cause for their condition.

The aim of this study was therefore to establish whether there is consensus among experienced ER neurologists about the essential elements of the NE in the unconscious patient, and how this consensus compares to the GCS, FOUR score, and published AAN Guidelines for medical students.

## 2 METHODS

An analysis of eight widely used neurology textbooks (Bender et al., 2012; Biller et al., 2011; Delank & Gehlen, 2015; Fuller, 2013; Hacke, 2016; Mattle & Mumenthaler, 2015; Posner et al., 2007; Urban, 2012), as well as two emergency textbooks, (Marx et al., 2013; Tintinalli et al., 2010) collectively revealed 38 different steps for the NE of the unconscious patient. We asked 31 practicing board‐certified neurologists with >2 years of experience in the examination of unconscious patients (23 neurologists from the Department of Neurology and Neuroscience, Medical Center, University of Freiburg, Germany; eight neurologists from other German clinics with an ED) to list which of these 38 steps they would use (prior to cerebral imaging) to examine an ER patient with acute unconsciousness that is not due to a known cause such as cardiac arrest or TBI. Participants were asked to use a four‐point scale to assess the level of importance of each step (adapted from [19]): 4 = should always be included; 3 = included at least 80% of the time; 2 = sometimes included, but <80%; 1 = almost never included. In accordance with reference (Moore & Chalk, 2009), ratings with an average >3 were ranked as “essential.” This process was facilitated by the Web‐based questionnaire system (http://www.umfrageonline.com).

Results were compared to those of the: (a) “Glasgow Coma Scale” (GCS) (Teasdale & Jennett, 1974), (b) “Full Outline of UnResponsiveness Score” (FOUR Score) (Wijdicks et al., 2005), and (c) “Guidelines for the Neurologic Examination in Patients with Altered Level of Consciousness” by the Neurology Clerkship Core Curriculum of the American Academy of Neurology (Gelb et al., 2002).

The study was approved by the local Ethics Committee (EK‐Freiburg No. 10003/18).

## 3 RESULTS

Twenty‐seven of 31 neurologists completed the survey. The results (mean ± standard deviation, *SD*) of the survey are shown for all 38 steps in Table 1. A total of 24 steps had a mean rating of 3.0 or higher. Notably, more examination steps were rated by the survey as essential when compared to the GCS and the FOUR score (Table 1). In contrast, almost all the recommended steps included in the “Guidelines for the Neurologic Examination in Patients with Altered Level of Consciousness” were rated by our experienced neurologists as essential steps of the NE; the exception here was caloric testing, which is sometimes used in intensive care units but is not commonplace in a German ER. On the other hand, highly rated items related to inspection steps (spontaneous position of the eyes, respiratory pattern), and testing for neck stiffness, mimic muscles, visual fixation and passive lifting of the extremities, are not included in the AAN Guidelines for medical students.

**Table 1:** Results of the Survey

	Item	Mean	*SD*	GCS	FOUR	Core curriculum AAN
1	**Spontaneous position of the eyes**	3.96	0.19			
2	**Pupillary light reflex**	3.89	0.42		x	x
3	**Response to auditory stimuli (including voice)**	3.89	0.42	x	x	x
4	**Spontaneous involuntary movements**	3.89	0.42		x	x
5	**Babinski reflex**	3.89	0.42			x
6	**Voluntary movements**	3.85	0.45		x	x
7	**Patellar reflex**	3.81	0.39			x
8	**Neck stiffness**	3.79	0.56			
9	**Tone, upper extremities**	3.78	0.50			x
10	**Biceps reflex**	3.78	0.57			x
11	**Nonmotor response to noxious stimuli (applied centrally, and to each limb individually)**	3.78	0.57	x		x
12	**Motor response to noxious stimuli (applied to each limb individually)**	3.74	0.70	x	x	x
13	**Mimic muscles**	3.69	0.77			
14	**Corneal reflex**	3.67	0.72		x	x
15	**Oculocephalic reflex**	3.63	0.67			x
16	**Visual fixation**	3.62	0.88		x	
17	**Tone, lower extremities**	3.59	0.78			x
18	**Visual testing/Response to visual threat**	3.44	0.87			x
19	**Respiratory pattern**	3.41	0.91		x	
20	**Achilles reflex**	3.41	0.91			x
21	**Reaction to passive lifting of arms**	3.37	0.91			
22	**Brachioradialis reflex**	3.31	0.95			x
23	**Reaction to passive lifting of legs**	3.19	1.02			
24	**Gag reflex**	3.11	0.92		x	x
25	Nonmotor response to noxious stimuli (applied to the face)	2.92	1.07			
26	Ankle clonus	2.70	1.21			
27	Triceps reflex	2.69	1.10			x
28	Other Plantar responses (Oppenheim, Gordon, Chaddock)	2.41	0.99			
29	Primitive reflexes	2.37	0.95			
30	Eyelid closing reflex	2.33	1.15			
31	Brudzinski's sign	2.31	1.03			
32	Lasegue's sign	2.12	0.97			
33	Kernig's sign	2.08	0.96			
34	Abdominal reflex	2.07	0.90			
35	Orbicularis sign	1.85	0.97			
36	Masseter reflex	1.70	0.85			
37	Ciliospinal reflex	1.67	0.77			
38	Fundoscopy	1.23	0.42			

Note

GCS: Glasgow coma scale; Core Curriculum AAN: “Guidelines for the Neurologic Examination in Patients with Altered Level of Consciousness” of the Neurology Clerkship Core Curriculum of the American Academy of Neurology; FOUR: Full Outline of UnResponsiveness; *SD*: standard deviation.

Items rated >3 are in bold.

## 4 DISCUSSION

The experienced neurologists who completed our survey identified 24 essential steps for the NE of the acutely‐unconscious patient. Five of these steps pertain to the inspection of the patient (Steps 1, 3, 4, 6, and 19). The inspection of movements allows important conclusions to be reached about the underlying cause of unconsciousness; side‐specific movements point to a structural cause, while subtle movements such as those occurring during a nonconvulsive state can be suggestive of an epileptic cause. An appraisal of the patient's breathing pattern can help distinguish between specific cerebral dysfunction or unspecific extra‐cerebral events as the potential underlying reasons for unconsciousness (Posner et al., 2007). Another important group of examination steps involving the brainstem (Steps 2, 13, 14, 15, 16, 18, and 24) are particularly informative due to their excellent localization potential and adjacency to the arousal system (Fuller, 2013). As computer tomography (CT) has a low sensitivity for ischemic brainstem lesions (Hwang, Silva, Furie, & Greer, 2012), the results may indicate normal brainstem structure despite severe ischemia. Therefore, subtle clinical changes in brainstem function might be the only clue for brainstem infarction, where subsequent application of CT‐angiography to detect basilar artery occlusion is the crucial step for initiating immediate thrombolysis or mechanical thrombectomy (Mak, Ho, Chan, Poon, & Wong, 2016). Moreover, the brainstem examination steps are important indicators for events relating to herniation (McNealy & Plum, 1962). Examination of the motor system (motor responses in Steps 9, 12, 17, 21, and 23, and reflex responses in Steps 5, 7, 10, 20, and 22) is also important for detecting posturing responses that are indicative of increased intracranial pressure or herniation processes, while simultaneously helping with the identification of asymmetric focal signs. The steps for examining the sensory system with noxious stimuli (Steps 8 and 11) facilitate the localization process and help uncover potential causes of meningeal irritation, such as meningitis or subarachnoid hemorrhage. Interestingly, testing both for neck stiffness – after careful exclusion of preceding trauma – and the Babinski sign are highly recommended by our experts. This reflects the importance of using highly specific tests (Isaza Jaramillo et al., 2014; Nakao, Jafri, Shah, & Newman, 2014; van de Beek et al., 2004) that recognize conditions requiring prompt initiation of treatment (Auburtin et al., 2006; Möhlenbruch et al., 2014), although mixed results regarding the sensitivity of each sign have been reported (Isaza Jaramillo et al., 2014; Mattle et al., 2011).

Despite being recommended in reviews about the approach to the comatose patient (e.g., Stevens & Bhardwaj, 2006; Stevens, Cadena, & Pineda, 2015), fundoscopy was rated lowest by our neurologists. Reasons are speculative only: As fundoscopy may be a useful examination step in the approach to the awake patient with acute headache in the ED to stratify further diagnostic steps (Sachdeva et al., 2018), its role for acute coma is unclear, as the development of papilledema as the most relevant finding in the comatose patient is usually to be expectable at least in the range of several hours and therefore may not be helpful in the clarification of the etiology of acute coma. Moreover, technical difficulties and time consumption of the examination itself, time‐consuming examination, and an overall low sensitivity for detecting papilledema in undilated pupils by nonophthalmologists may also play important roles. The availability of new devices, like nonmydriatic cameras (Thulasi, Fraser, Biousse, Wright, Newman & Bruce, 2013), may be a way to overcome the technical barriers, but more data are needed about the diagnostic value in this specific patient population.

Despite the seemingly high number of steps, the resulting examination can be completed within 2–3 min in the hands of an experienced examiner, particularly since five of these steps are observational only. Moreover, depending on the individual situation, not all 24 steps need to be performed every time (e.g., not all five deep tendon reflexes are performed in febrile patients with suspected meningitis).

It is interesting to note that experienced neurologists apply considerably more examination steps in comparison with the “Glasgow Coma Scale” (GCS) (Teasdale & Jennett, 1974), or the “Full Outline of UnResponsiveness Score” (FOUR Score) (Wijdicks et al., 2005), which are often used by emergency physicians. However, both these coma scoring methods were designed to predict the outcome of comatose patients rather than to help diagnose the underlying condition of the coma. Although it is not clear whether the extra steps rated by our experienced neurologists further benefit the diagnostic process – and hence improve patient outcome – the results of this survey may serve as a useful basis for future studies comparing the use of short scales by emergency physicians to the essential neurological examination recommended (and performed) by neurologists.

In contrast, the “Guidelines for the Neurologic Examination in Patients with Altered Level of Consciousness” by the Neurology Clerkship Core Curriculum of the American Academy of Neurology (Gelb et al., 2002) matched noticeably well with the practical approach of our experienced neurologists: Almost all the recommended steps in the guidelines were included in the items rated as essential in our survey. As the voting process for these guidelines is not described in detail, it remains unclear whether they are based on practical experience or theoretical considerations. In any case, our survey of experienced neurologists validates the use of this curriculum as a solid basis for teaching medical students (and also nonneurologists) the NE of the unconscious patient. However, it needs to be reconsidered whether the seven additionally recommended steps missing in the guidelines should be included, as they are all rather noncomplex steps and hence easily teachable.

The limitations of this study include the restricted number of participants. The majority of neurologists came from one university; however, seven of the 23 neurologists from the Department of Neurology and Neuroscience of the University of Freiburg completed their residency in the neurology departments of other university hospitals in Germany and Switzerland. Hence, almost half of the asked neurologists (15 of 31) were trained outside the Department of Neurology and Neuroscience of the University of Freiburg. Due to the anonymous character of the questionnaire, we were not able to find out the distribution in the de facto completed questionnaires. In addition, although the use of the Delphi method (de Villiers, de Villiers, & Kent, 2005) could have strengthened the results, the standard deviations of the vast majority of examination steps achieved by single questioning were comparable to those of Moore et al., who did apply the Delphi method (Moore & Chalk, 2009). All of these limitations could be overcome by repeating this study at other clinical locations, as conducted for the “essential neurological examination” (Lima & Maranhão‐Filho, 2012; Moore & Chalk, 2009). Another limitation relates to the method of selecting the steps from a given list, which might result in a higher number of essential steps compared to actively listing the steps used. Moreover, depending on the individual scenario, adjustments to the number of applied steps might also occur.

In summary, we present the first data on the essential components of the NE in the unconscious patient, as generated by neurologists with long‐term experience in the ER. These results could serve to validate the particular components of the NE of unconscious patients that expert neurologists consider important and may help to focus on teaching the most important examination steps to medical students and non‐neurologists working in emergency departments.

## CONFLICT OF INTEREST

None of the authors declare conflict of interests.
